# Robust Precoding for Multi-User Visible Light Communications with Quantized Channel Information

**DOI:** 10.3390/s22239238

**Published:** 2022-11-28

**Authors:** Olga Muñoz, Antonio Pascual-Iserte, Guillermo San Arranz

**Affiliations:** Department of Signal Theory and Communications, Universitat Politècnica de Catalunya, 08034 Barcelona, Spain

**Keywords:** visible light communications, robust precoding, channel state information, quantization, convex optimization

## Abstract

In this paper, we address the design of multi-user multiple-input single-output (MU-MISO) precoders for indoor visible light communication (VLC) systems. The goal is to minimize the transmitted optical power per light emitting diode (LED) under imperfect channel state information (CSI) at the transmitter side. Robust precoders for imperfect CSI available in the literature include noisy and outdated channel estimation cases. However, to the best of our knowledge, no work has considered adding robustness against channel quantization. In this paper, we fill this gap by addressing the case of imperfect CSI due to the quantization of VLC channels. We model the quantization errors in the CSI through polyhedric uncertainty regions. For polyhedric uncertainty regions and positive real channels, as is the case of VLC channels, we show that the robust precoder against channel quantization errors that minimizes the transmitted optical power while guaranteeing a target signal to noise plus interference ratio (SNIR) per user is the solution of a second order cone programming (SOCP) problem. Finally, we evaluate its performance under different quantization levels through numerical simulations.

## 1. Introduction

### 1.1. Background and Motivation

In the past years, researchers have proposed different multi-user precoding techniques for the visible light communication (VLC) channel, assuming the availability of channel state information (CSI) at the transmitter side. In radio frequency (RF) systems, there are methods to acquire the CSI, such as those based on the exploitation of channel reciprocity between uplink (UL) and downlink (DL) in time division duplexing (TDD) or the use of a feedback channel in frequency division duplexing (FDD). Unfortunately, establishing an UL feedback channel in VLC is not straightforward. One possibility, which does not relay on channel reciprocity, is to send the UL signals through an RF channel. In this case, once the receivers have estimated the DL channel, the estimates can be fed back to the transmitter through the RF UL link. Consequently, the CSI available at the transmitter side will be far from being accurate, as it can be noisy, outdated, and will contain quantization errors.

In this paper, we address the design of a multi-user multiple-input single-output (MU-MISO) precoder robust against channel quantization errors, which will be the dominant source of error in the CSI if the signal to noise ratio (SNR) during the channel estimation is high or if the number of quantization bits is low. We model the quantization errors in the CSI through polyhedric uncertainty regions that contain the actual channel. Interestingly, for polyhedric uncertainty regions and the fact that, for VLC communications, the transmitted signals and the channels are positive real magnitudes allows us obtaining a non-approximated solution for the robust precoder. This design has not been developed before for RF or VLC channels. In particular, for VLC channels, the only available solutions are those considering estimation noise or outdated CSI, which do not fit the model corresponding to quantization errors, as explained in what follows.

### 1.2. Related Work

In Ref. [1], the authors consider a MU-MISO VLC system in a scenario where the passengers in a train wagon receive data through the light emitting diodes (LEDs) placed at the ceiling. Similarly, in Refs. [2,3,4,5,6] the authors address the design of the DL in VLC systems under the perfect CSI assumption. In this paper, as mentioned before, we consider a MU-MISO scenario similar to those considered in the previous references (and, in particular, in Ref. [1]) but assuming that the CSI is not perfect. The scenario is summarized in Section 2 for completeness.

Imperfect CSI has been considered in Refs. [7,8,9,10]. The authors of Refs. [7,8] model the channel error as zero-mean Gaussian distributed with a certain covariance matrix to account for noisy channel estimation. The authors of Ref. [9] impose an upper bound on the Frobenius norm of the channel error to account for outdated CSI. Finally, the authors of Ref. [10] consider both the stochastic and bounded error model to account for both channel estimation errors and outdated CSI. None of these papers [7,8,9,10], however, have considered channel estimation errors due to the quantization of the channel response. In this paper, we fill this gap by considering imperfect CSI due to the quantization of VLC channels. Note that, in the absence of channel reciprocity, for example if the UL cannot be optical, the receiver needs to report the channel response through a digital feedback channel. This makes quantization a primary CSI error source, particularly when the channel estimation SNR is good or when the number of quantization bits is low.

Finally, regarding the precoder designs, some authors design zero forcing (ZF) linear precoders while maximizing the weighted sum rate or other figures of merit, e.g., [1,6]. Other works do not relay on ZF and, instead, focus on minimizing the global mean square error (MSE), e.g., [7], or the average MSE and the worst-case MSE, e.g., [10], for the noisy CSI and the outdated CSI, respectively. Similarly, in Refs. [8,9], the authors address the maximization of the minimum signal to noise plus interference ratio (SNIR), also for the cases of noisy and outdated CSI, respectively. In our work, instead of ZF precoders as in Refs. [1,6], we minimize the optical power per LED subject to a target SNIR per user without forcing null interference, which makes the design more versatile, and account explicitly for quantization errors. On the other hand, instead of considering a global figure of merit that cannot guarantee the quality of individual users, such as the global or the average MSE, we deal with individual user SNIR constraints.

### 1.3. Main Contributions

Our main contributions and novelties in this paper are the following:

- The design of a robust MU-MISO precoder for VLC systems under imperfect CSI at the transmitter due to channel quantization. We show that for VLC with CSI imperfections due to quantization, the robust precoder (optimum in terms of transmitted optical power per LED) is the solution of a second order cone programming (SOCP) problem [11];
- Quantization guidelines and discussion of their impact on the beamforming design;
- The evaluation of the performance of the robust precoder under different levels of quantization.

## 2. System Model

We consider a VLC system, where a transmitter equipped with *L* LEDs communicates simultaneously with *K* users (with indexes k=1,…,K) deployed in the same room.

The transmitter modulates the intensity of the light emitted by each LED according to the information symbols. Let $s={\left(s_{1},s_{2},\ldots ,s_{K}\right)}^{T}\in R^{K\times 1}$ be the vector containing the information-bearing real symbols, one per user, at time *t*, although the time index *t* is omitted for simplification. We model, without loss of generality, each symbol as a random variable (r.v.) taken from an alphabet with zero mean $E\{s_{k}\}=0$ and unit variance $E\{s_{k}^{2}\}=1$. We assume that the dynamic range of each symbol is limited so that $-A_{k}\leq s_{k}\leq A_{k}$.

Using a set of real beamvectors $w_{k}\in R^{L\times 1}$, k=1,…,K, the transmitter combines the set of transmitted symbols at time *t*, ${\left\{s_{k}\right\}}_{k=1}^{K}$. Therefore, the vector $x\in R^{L\times 1}$ containing the *K* optical transmitted symbols at time *t* (omitted again for simplification) can be written as

(1) $$x=\sum_{i=1}^{K}w_{i}s_{i}+\beta 1\in R_{+}^{L\times 1},$$

where β is the direct current (DC) component equal to the average optical power emitted per LED. Each component of the vector x, at any given instant *t*, corresponds to the optical transmit power per individual LED, which is a positive physical magnitude and is usually limited due the technological constraints and eye safety reasons. Therefore, the set of precoders ${\left\{w_{k}\right\}}_{k=1}^{K}$ should be designed to guarantee that, for any combination of symbols, every component of x is positive and below the maximum allowed optical power, $P_{\max}$. On the other hand, β determines the average light intensity. Because of this, in practice, β is a preset value, equal for all the LEDs, and we will assume so in this work.

At the receiver side, each user is equipped with a single photo diode (PD) that provides an electrical signal according to the incident light and the responsivity ρ of the PD (i.e., the ratio of the output photocurrent to the input optical power measured in [A/W]). The incident light depends on the light emitted by the set of LEDs and the channel gain between the *l*-th LED and the *k*-th PD denoted by $h_{k,l}\in R_{+}$ (a magnitude which is, again, real and positive). The channel gain, $h_{k,l}$, depends on the distance between the *l*-th LED and the *k*-th PD, the incident angle, and the irradiation angle, as well as other parameters (see [1] for a full description). The column vector containing all the channel gains from all the LEDs to the *k*-th user’s PD is given by $h_{k}={\left(h_{k,1},h_{k,2},\ldots ,h_{k,L}\right)}^{T}\in R_{+}^{L\times 1}$.

Accordingly, the electrical signal at the output of the *k*-th PD, $y_{k}\in R$, is equal to

(2) $$y_{k}=\rho h_{k}^{T}x+n_{k}=\rho h_{k}^{T}\sum_{i=1}^{K}w_{i}s_{i}+\rho \beta h_{k}^{T}1+n_{k},$$

where $n_{k}$ is the real-valued additive white Gaussian noise with zero mean and variance $\sigma_{k}^{2}$ modeling the thermal and shot noise [12]. The term $\beta h_{k}^{T}1$ is a DC component that can be easily estimated and removed by the receiver as it does not provide any information about the transmitted symbols. After removing the DC component, the electrical SNIR at the *k*-th PD can be written as (note that the receiver converts the optical power to current according to the responsivity factor ρ, which implies that the receiver electrical SNR in VLC is proportional to the square of the received optical average power, while in RF it is directly proportional to the received average power, as explained in Ref. [1]):

(3) $${\mathrm{SNIR}}_{k}=\frac{\rho^{2}{\left|h_{k}^{T}w_{k}\right|}^{2}}{\sigma_{k}^{2}+\sum_{i\neq k}\rho^{2}{\left|h_{k}^{T}w_{i}\right|}^{2}}.$$

The symbol error rate (SER) depends on the SNIR. This means that, if a minimum detection quality in terms of a maximum SER is required, this can be translated equivalently into a minimum SNIR requirement per user denoted by $\gamma_{k}$. Note that the relation between the maximum SER and $\gamma_{k}$ depends on the size of the adopted modulation strategy (e.g., *M*-PAM), that is, the transmission rate [12]. If different users use different modulations, the corresponding minimum required SNIRs will also differ.

## 3. Problem Formulation

In this section, we address the design of a robust MU-MISO precoder under imperfect CSI at the transmitter side for the VLC system described Section 2. We assume that the receivers report the DL channels to the transmitter through a feedback channel. Accordingly, the informed channels may differ from the actual channels due to estimation and reporting errors:

- If the source of CSI imperfection is the quantization, the actual channel will be within an uncertainty region whose shape and size depend on the quantization type (scalar or vector quantization) and the number of quantization bits;
- If the source of imperfection is an outdated CSI, the effect can be modeled by imposing an upper bound on the Frobenius norm of the channel error, as in Refs. [9,10]. In this case, the actual channel will be within a spherical uncertainty region;
- If the source of CSI imperfection is the channel estimation noise (assumed as Gaussian in Refs. [7,8]), the norm of the error in the CSI will be upper-bounded by a threshold with a certain probability $p_{in}<1$ and, therefore, the corresponding uncertainty region will be spherical. The value of the threshold depends on the estimation SNR and the value of $p_{in}$.

If the three previous sources for CSI imperfection are simultaneously present, the global uncertainty region is a convolutive combination of the different regions [13]. In this paper, we do not consider the channel estimation noise and the outdated channel information and assume that the quantization effect is dominant, which is a valid assumption in the case of having good channel estimation SNR or a low number of quantization bits and when the channel does not vary quickly. For simplicity, we also assume that each receiver *k* quantizes the acquired channel response $h_{k}$ independently.

For a channel quantizer based on *vector quantization* and characterized by a set of *N* reconstruction points $\left\{c_{i}\in R^{L\times 1},i=1,\ldots ,N\right\}$, the uncertainty region associated to each reconstruction point (or centroid) $c_{i}$ is defined as:

(4) $$R_{i}=\{h\in R_{+}^{L\times 1}\;|\;||h-c_{i}\left|\right|\leq ||h-c_{j}||\;\forall j\neq i\}.$$

The centroids ${\left\{c_{i}\right\}}_{i=1}^{N}$ correspond to the *N* codewords composing the quantizer codebook. Therefore, each receiver requires sending ceil$\left(\log_{2}\left(N\right)\right)$ bits for CSI feedback. Note that $R_{i}$ is the Voronoi partition around the centroid $c_{i}$ with respect to (w.r.t.) the set ${\left\{c_{i}\right\}}_{i=1}^{N}$. As the space is a finite-dimensional Euclidean space, these Voronoi cells are convex polygons completely defined by their vertices [11], that is, polyhedric uncertainty regions.

The previous notation encompasses, as a particular case, *scalar quantization*, that is, an independent quantization of each component of the channel vector. In this particular case, the uncertainty regions are rectangular and the number of vertices of each region is $2^{L}$.

In what follows, we refer to the uncertainty region of the channel informed by the *k*-th user as $H_{k}$. For the *k*-th user, $H_{k}$ will be one of the Voronoi regions within the set $\left\{R_{1},\ldots ,R_{N}\right\}$. Each region is a convex polygon that can be expressed as the convex hull [11] of its $J_{k}$ vertices $h_{k}^{\left(j\right)},j=1,\ldots ,J_{k}$, that is: $H_{k}=\mathrm{coh}\left(\left\{h_{k}^{\left(j\right)},j=1,\ldots ,J_{k}\right\}\right)$.

Having defined the uncertainty regions, in the following we formulate the design of a precoder fulfilling the SNIR constraint per user for any possible channel in the uncertainty region:

(5) $$\begin{array}{cc} \underset{\{w_{k}\},v}{\mathrm{minimize}\;}\; & v\\ \mathrm{subject}\;\mathrm{to}\;\; & C1:\frac{\rho^{2}{\left|h_{k}^{T}w_{k}\right|}^{2}}{\sigma_{k}^{2}+\sum_{i\neq k}\rho^{2}{\left|h_{k}^{T}w_{i}\right|}^{2}}\geq \gamma_{k},\;\;\forall h_{k}\in H_{k},\;k=1,\ldots ,K;\\ \; & C2:\sum_{k}A_{k}\left|e_{l}^{T}w_{k}\right|\leq v,\;\;l=1,\ldots ,L;\\ \; & C3:0\leq v\leq \min(\beta ,P_{\max}-\beta ). \end{array}$$

The C1 constraints impose the *K* minimum SNIR requirements (one per user). In C2, $e_{l}={\left(0,\ldots ,0,1,0,\ldots ,0\right)}^{T}$ is a vector whose *l*-th element is equal to 1, and the rest of elements are zero. The C2 constraints imply that the optical power transmitted per LED is within the interval [β−v,β+v]. The C3 constraint ensures that [β−v,β+v] is within the interval $\left[0,P_{\max}\right]$, and, therefore, the optical power transmitted per LED is positive and not greater than $P_{\max}$.

With some basic manipulations, we can re-write the C1 constraints as follows:

(6) $$C1:\sqrt{\sigma_{k}^{2}+\sum_{i\neq k}\rho^{2}{\left|h_{k}^{T}w_{i}\right|}^{2}}\leq \frac{\rho }{\sqrt{\gamma_{k}}}\left|h_{k}^{T}w_{k}\right|,\;\forall h_{k}\in H_{k},\;k=1,\ldots ,K.$$

Both alternative expressions of constraints C1 (either in (5) or in (6)) represent *K* sets of infinite non-convex constraints because the constraint expressed in C1 for each *k* has to be fulfilled for the infinite set of channels represented by $H_{k}$.

## 4. Solution for Polyhedric Uncertainty Regions

In this section, we show that, for polyhedric uncertainty regions $H_{k}$, problem (5) can be re-formulated as a convex problem with a finite number of second-order cone constraints. Therefore, the problem becomes a SOCP problem, for which several efficient interior point methods are available [11].

As a first step, we show in Lemma 1 that we can force, without any loss of generality, $w_{k}^{T}h_{k}$ to be positive for any channel within the region $H_{k}$.

**Lemma 1.**  *Any feasible precoder $w_{k}$ will be such that $w_{k}^{T}h_{k}$ will be always positive or always negative for any $h_{k}\in H_{k}$.*

**Proof.**  We will prove it by contradiction. Let us assume that there is a feasible $w_{k}$, that is, a vector $w_{k}$ that satisfies constraints C1, C2, and C3, and two channels in $H_{k}$, namely $h_{k}^{\left(1\right)},h_{k}^{\left(2\right)}\in H_{k}$, such that $w_{k}^{T}h_{k}^{\left(1\right)}>0$ and $w_{k}^{T}h_{k}^{\left(2\right)}<0$. Therefore, we can find α such that $w_{k}^{T}{\bar{h}}_{k}=0$ with ${\bar{h}}_{k}=\alpha h_{k}^{\left(1\right)}+\left(1-\alpha \right)h_{k}^{\left(2\right)}$ and 0<α<1. In fact, we have:

(7) $$\alpha =\frac{-w_{k}^{T}h_{k}^{\left(2\right)}}{w_{k}^{T}h_{k}^{\left(1\right)}-w_{k}^{T}h_{k}^{\left(2\right)}}=\frac{|w_{k}^{T}h_{k}^{\left(2\right)}|}{|w_{k}^{T}h_{k}^{\left(1\right)}\left|+\right|w_{k}^{T}h_{k}^{\left(2\right)}|}.$$

As $H_{k}$ is convex, such ${\bar{h}}_{k}\in H_{k}$. Therefore, due to the noise term, C1 is not fulfilled, and consequently, $w_{k}$ cannot be a feasible solution. □

As $w_{k}^{T}h_{k}$ is either always negative or always positive for all $h_{k}\in H_{k}$ for a feasible value of $w_{k}$, we can impose, without loss of generality, that $w_{k}^{T}h_{k}>0$. Note that if the optimum precoder, $w_{k}^{⋆}$, was such that ${w_{k}^{⋆}}^{T}h_{k}$ was negative, we could multiply $w_{k}^{⋆}$ by (−1) without changing the values of the objective and constraint functions. Imposing strict positivity for any $h_{k}\in H_{k}$ allows us to remove the absolute value in the right-hand side of C1 in (6). After removing that absolute value, we have *K* infinite sets of convex constraints, because a convex constraint has to be fulfilled for a infinite set of channels $h_{k}\in H_{k}$ with k=1,…,K. To deal with this complexity, consider now the following lemma.

**Lemma 2.**  *Let us take a convex region formulated as $H_{k}=coh\left(\left\{h_{k}^{\left(j\right)},j=1,\ldots ,J_{k}\right\}\right)$ and a function $f_{w_{k}}\left(h_{k}\right)$ parameterized by $w_{k}$ and convex w.r.t. $h_{k}$. If $f_{w_{k}}\left(h_{k}^{\left(j\right)}\right)\leq 0$, $j=1,\ldots ,J_{k}$, then $f_{w_{k}}\left(h_{k}\right)\leq 0,\forall h_{k}\in H_{k}$.*

**Proof.**  As the uncertainty region $H_{k}$ is the convex hull of their $J_{k}$ vertices, any element in $H_{k}$ can be expressed as a linear convex combination of their vertices:

(8) $$h_{k}=\sum_{j=1}^{J_{k}}\alpha_{j}h_{k}^{\left(j\right)},\;\alpha_{j}>0,\;\sum_{j=1}^{J_{k}}\alpha_{j}=1.$$

Consider now the following function

(9) $$f_{w_{k}}\left(h_{k}\right)=\sqrt{\sigma_{k}^{2}+\sum_{i\neq k}\rho^{2}{\left(h_{k}^{T}w_{i}\right)}^{2}}-\frac{\rho }{\sqrt{\gamma_{k}}}h_{k}^{T}w_{k}$$

that is convex w.r.t. $h_{k}$. Due to its convexity, it fulfills:

(10) $$f_{w_{k}}\left(h_{k}\right)=f_{w_{k}}\left(\sum_{j=1}^{J_{k}}\alpha_{j}h_{k}^{\left(j\right)}\right)\leq \sum_{j=1}^{J_{k}}\alpha_{j}f_{w_{k}}\left(h_{k}^{\left(j\right)}\right).$$

Therefore, if $f_{w_{k}}\left(h_{k}\right)\leq 0$ for all the vertices $\left\{h_{k}^{\left(j\right)}\right\}$, then $f_{w_{k}}\left(h_{k}\right)$ will be less than or equal to 0 for all $h_{k}\in H_{k}$. □

Thanks to Lemma 2, we can re-write problem (5) as:

(11) $$\begin{array}{cc} \underset{\{w_{k}\},v}{\mathrm{minimize}\;}\; & v\\ \mathrm{subject}\;\mathrm{to}\;\; & C1:\sqrt{\sigma_{k}^{2}+\sum_{i\neq k}\rho^{2}{\left|w_{i}^{T}h_{k}^{\left(j\right)}\right|}^{2}}\leq \frac{\rho }{\sqrt{\gamma_{k}}}w_{k}^{T}h_{k}^{\left(j\right)},\;j=1,\ldots ,J_{k},\;k=1,\ldots ,K,\\ \; & C2:\sum_{k}A_{k}\left|e_{l}^{T}w_{k}\right|\leq v,\;l=1,\ldots ,L,\\ & C3:0\leq v\leq \min(\beta ,P_{\max}-\beta ),\\ \; & C4:w_{k}^{T}h_{k}^{\left(j\right)}\geq 0,\;j=1,\ldots ,J_{k},\;k=1,\ldots ,K. \end{array}$$

Note that we have been able to ensure the positiveness of $w_{k}^{T}h_{k}$ in the whole uncertainty region by just forcing it at its vertices through the finite set of $\sum_{k=1}^{K}k_{i}$ convex constraints in C4. As a result, the original problem turns out into a SOCP with a finite number of constraints for which several extremely efficient algorithms and tools are available, such as the interior point methods and the SeDuMi software package [11,14].

A problem similar to (5) was considered in Ref. [15] (Section 18.5.1) for RF MU-MISO channels, for which channel and beamformers are complex-valued vectors. First, the authors considered perfect CSI and solved the problem by forcing the imaginary part of $w_{k}^{T}h_{k}$ to be zero and the real part to be positive (Equation (18.29) in [15]). Then, they extended the solution to a robust scheme modeling the uncertainty through the lower and upper bounds of the channel correlation matrix (Equation (18.4) in Ref. [15]). Note that this model does not fit the case where the channel uncertainty comes from quantization, which results in uncertainty regions different from the ones considered in Ref. [15]. In addition, the strategy followed in Ref. [15] for the perfect CSI case cannot be directly applied when having an infinite set of channels (a region) in the constraints instead of a single channel. For RF channels, unless all the channels are 0, it is not possible to ensure a null imaginary part (and a positive real part) of $w_{k}^{T}h_{k}$ for all the channels in the region. Fortunately, in VLC systems, the channels do not have imaginary parts, and we have proved in Lemma 1 that the real part of $w_{k}^{T}h_{k}$ cannot change its sign within the whole uncertainty region.

## 5. Implementation Aspects

A practical implementation of the proposed scheme requires each user to estimate the DL channel from each LED and send a quantized version of this information through a feedback channel. In this section, we discuss some issues regarding the quantization and the impact on the complexity of the optimization problem presented in Section 3 and Section 4.

The most straightforward quantization strategy is to independently quantize the *L* channel components (*scalar quantization*) using a uniform quantizer of *B* bits per channel component. However, depending on the channel amplitudes distribution, some strategies can be taken to reduce the quantization error power without increasing the number of bits. For example, if lower amplitudes are more likely than higher amplitudes, more efficient usage of the available representation levels is achieved if a non-uniform quantizer is employed (for example, a uniform quantizer of the logarithm of the channel).

For uniform or non-uniform scalar quantization with *B* bits per channel component, the number of possible uncertainty regions is $N=2^{L\cdot B}$. As any uncertainty region reported has $2^{L}$ vertices, the total number of constraints represented by C1, $K\cdot 2^{L}$, increases linearly with *K* and exponentially with *L*.

On the other hand, in the scenario considered, some combinations of the amplitudes of the LEDs are not possible. For example, as each LED location is different, a user cannot have a maximum channel simultaneously from all LEDs. A joint quantization of channel components (*vector quantization*) can exploit this feature to reduce the number of CSI information bits. The total number of constraints will depend again on the number of vertices of the uncertainty regions. Nevertheless, the vector quantization case is outside the scope of this paper and we will restrict the simulations to the scalar quantization case.

## 6. Simulation Results

**Figure 1:**
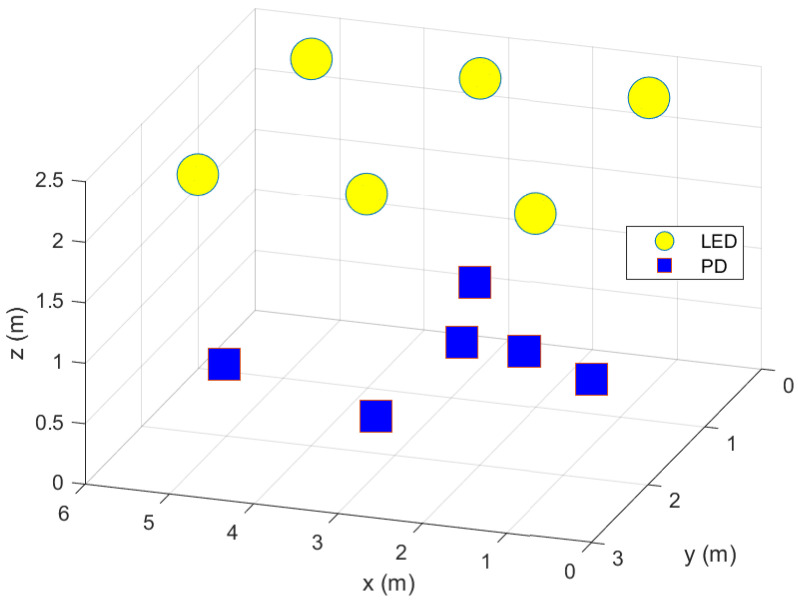
Distribution of light emitting diodes (LEDs) (fixed positions) and photo diodes (PDs) (random positions) in a 3D space.

In this section, we present some simulation results. We consider L=6 active LEDs serving *K* users (see Figure 1 as an example of the simulated setup, similar to the one considered in Ref. [1]). We set the target SNIR to 15 dB for each user, the maximum power threshold to 20 W, and take the rest of the parameters from Ref. [1]. All the simulation results presented in this section are averaged over different scenario realizations. For each scenario realization, the LEDs are positioned as shown in Figure 1 with a fixed height of 2.4 m. The users’ (PDs) locations are random. The *x* and *y* coordinates of the PDs are drawn from a uniform random distribution in the simulated area, whereas the *z* coordinate (height of PD) is drawn from a uniform random distribution between 0.5 and 1 m. The channels are then generated according to the relative positions between transmitters (LEDs) and receivers (PDs).

For each scenario realization, we design precoders that serve *K* users jointly. The quantized channel is the only CSI available at the transmitter side to design robust and non-robust precoders. For the simulations, we have considered the particular case of independent quantization of the channel between each LED and each PD, that is, scalar quantization. Each of these channel coefficients is a real positive magnitude that can take a wide margin of values. To cope with this wide margin, we perform a logarithm transformation and then a uniform quantization. In other words, we consider a uniform quantization of the value in dB of the channel magnitude. The dynamic margin of the quantizer, i.e., the minimum and maximum values represented by the quantizer, are determined statistically using $10^{6}$ realizations of users’ positions and channel values of the setup considered in the simulations. We design the non-robust precoder using the quantized channel as if it were the actual channel (despite being different). The actual channel is within an uncertainty region around the quantized channel. As explained in the previous sections, we design the robust precoder to achieve the target SNIR for any channel within this region. For the computation of the non-robust and robust solutions, we have used CVX, a package for specifying and solving convex programs [16,17].

**Figure 2:**
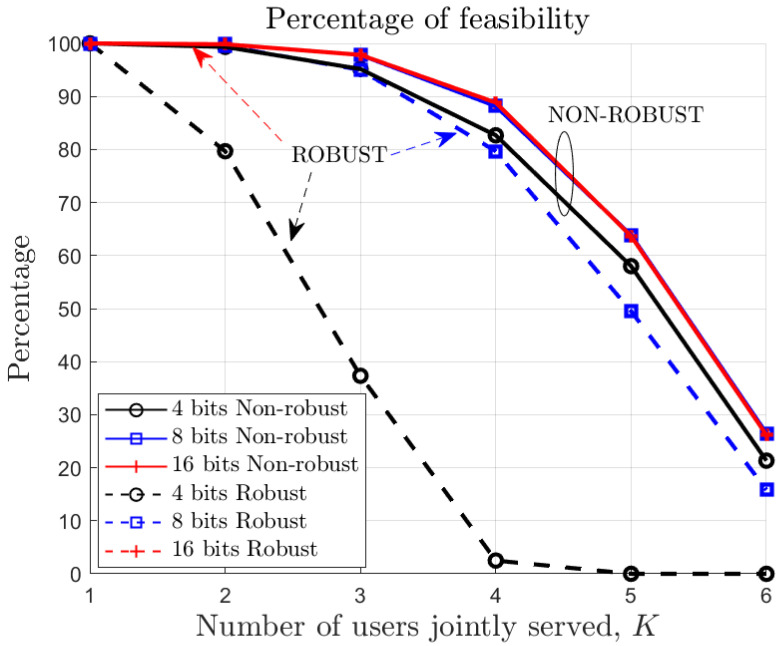
Percentage of successful designs (averaged over different channel realizations) for different numbers of jointly served users and quantization bits.

The number of bits impacts the size of the channel uncertainty regions, making the constraints of the robust precoder design problem more challenging. As a result, a higher optical power is needed to fulfill the constraints. Note that, depending on the channel realizations, fulfilling the constraints may require an unacceptable amount of power or not be possible at all; that is, it is not possible to fulfill constraint C3. In Figure 2, we show the percentage of feasibility (i.e., successful designs) for different numbers of jointly served users and quantization bits. As expected, the percentage reduces (i.e., the precoder design becomes more complex) as the number of bits decreases or as the number of users increases.

**Figure 3:**
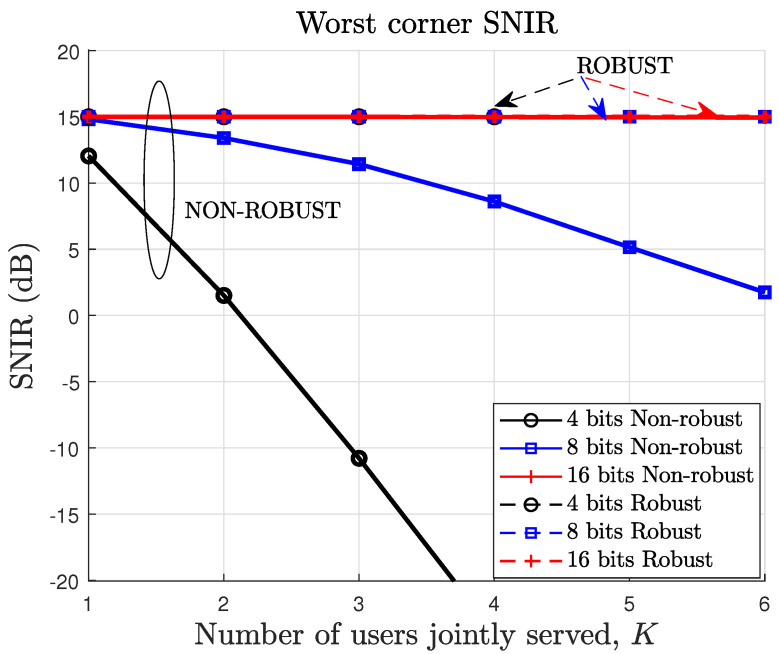
Signal to noise plus interference ratio (SNIR) at the worst corner of the uncertainty regions of the *K* users group (averaged over different channel realizations). The target SNIR is 15 dB.

It is not surprising that the percentage of feasibility cases of the robust design is lower than for the non-robust design due to the much harder constraints of the robust approach. Note, however, that the non-robust solution will perform much worse than the robust counterpart for channels different from the quantized version. To illustrate this, Figure 3 shows the SNIR for the worst possible channel, which always corresponds to a corner of the uncertainty region ($h_{k}^{\left(j\right)},j=1,\ldots ,J_{k}$, k=1,…,K of constraint C1), as proved in this paper, for the non-robust and robust approaches. Therefore, if a feasible solution exists, the robust precoder fulfills the SNIR constraint with equality at this corner by design, which is set to 15 dB in these simulations, and, consequently, in all of the uncertainty region. As a result, as shown in Figure 3, for the robust precoder, the SNIR at the worst-possible channel of the uncertainty region is always equal to the target SNIR. In contrast, the non-robust design cannot guarantee that, especially when the number of quantization bits is low.

**Figure 4:**
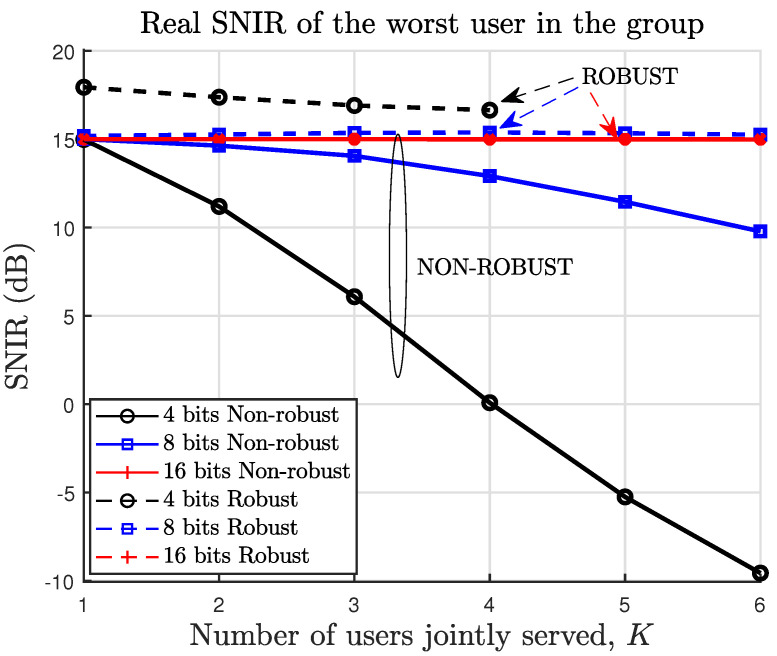
SNIR of the worst user within each group of *K* users (averaged over different channel realizations). The target SNIR is 15 dB.

In practice, we may be interested in finding how well the feasible designs will perform for the real channels experienced by the users. To that end, we compute the SNIR using the actual channels and the precoders designed using the quantized channels (as the actual channels information is not available at the design phase). In Figure 4, we show the performance of the robust and non-robust precoders in terms of the actual SNIR of the worst user within each group of *K* users, averaged over different channel realizations. The actual SNIR is the SNIR computed with the actual channels, which are different to the quantized channels used for the beamformer design. For the non-robust precoder, the achieved SNIR is below the target SNIR. This is because the non-robust design incorrectly assumes that the quantized channel is equal to the actual channel. On the other hand, the robust precoder is designed to achieve the target SNIR for any channel within the uncertainty region. The actual channel always lies within the uncertainty region. However, it is not necessarily the worst-case channel, which is located at a corner of the uncertainty region, as shown in previous sections. Consequently, with the robust beamformer, the actual users’ SNIR can be above the target SNIR, particularly if the uncertainty region increases, which happens when the number of quantization bits decreases. As illustrated previously in Figure 2, for 4 bits and 5 users or more, no robust precoder can achieve the target SNIR with a power lower than or equal to $P_{\max}$. This is the reason why no SNIR values are shown for these cases in Figure 3 and Figure 4. Note that this does not invalidate our approach. It just tells us that for 5 users or more, we need to increase the number of bits to fulfill the target SNIR under channel uncertainty with a power lower than or equal to $P_{\max}$.

To ensure feasibility, given the number of bits *B*, we can reduce the number of simultaneously served users *K* until a feasible precoder can be found. Moreover, as proved in Section 4, we can guarantee that the robust precoder will fulfill the target quality for all the users in the set. Table 1 shows the SNIR of the worst user within each set of jointly served users (with the maximum number of users possible provided that a feasible solution can be found). Note that, in the case of the non-robust precoder, the target quality cannot be achieved. More importantly, it is impossible to decide beforehand if the non-robust precoders will or will not do the job because only the quantized channel information (and not the actual channel) is available at the design stage.

## 7. Conclusions and Discussion

In this paper, we have designed the optimum MU-MISO precoder robust against channel quantization errors in VLC systems that minimizes the transmitted optical power while guaranteeing a target SNIR per user. We show that the exact form for this precoder can be obtained as the solution of an SOCP problem. As expected, the precoder design becomes more difficult (i.e., requires more power) as the number of bits decreases or as the number of users increases. Nevertheless, provided the budget power is sufficient, we can guarantee that the robust designs achieve the target SNIR per user. In contrast, for non-robust designs, such a guarantee is not possible.

As future work, we will consider the robust precoding design problem in the presence of both quantization noise and channel estimation errors. This could include also the case of vector quantization as a way to improve the quality of the CSI without increasing the number of quantization bits. Another research line will be the extension of the proposed scheme to the multiple-input multiple-outpout (MIMO) case, where each receiver has more than one PD. Finally, and given the non-linear responses of the LEDs and the PDs, it will be convenient to study how these non-linearities affect the proposed designs and analyze how to modify the designs to be robust also against them.

## 8. Materials and Methods

The results in this paper can be replicated by implementing the algorithms described in it. The authors disclose that the code is protected and cannot be made public. Interested readers can get in touch with the authors to receive assistance.

## Figures and Tables

**Table 1:** Real SNIR (dB) of the worst user within each feasible group (averaged over different channel realizations).

Number of Bits	B=4	B=8	B=16
Non-robust design	−3.5459	11.3752	14.9915
Robust design	17.3103	15.3762	15.0025

## Data Availability

Not applicable.

## Abbreviations

The following abbreviations are used in this manuscript:

CSI	Channel state information
DC	Direct current
DL	Downlink
FDD	Frequency division duplexing
LED	Light emitting diode
MIMO	Multiple-input multiple-outpout
MSE	Mean square error
MU-MISO	Multi-user multiple-input single-output
PD	Photo diode
RF	Radio frequency
SER	Symbol error rate
SNIR	Signal to noise plus interference ratio
SNR	Signal to noise ratio
SOCP	Second order cone programming
TDD	Time division duplexing
UL	Uplink
VLC	Visible light communication
w.r.t.	with respect to
ZF	Zero forcing

## References

[1] Viñals R., Muñoz O., Agustín A., Vidal J. Cooperative Linear Precoding for Multi-User MISO Visible Light Communications. Proceedings of the GLOBECOM 2017—2017 IEEE Global Communications Conference.

[2] Li B., Wang J., Zhang R., Shen H., Zhao C., Hanzo L. (2015). Multiuser MISO Transceiver Design for Indoor Downlink Visible Light Communication Under Per-LED Optical Power Constraints. IEEE Photonics J..

[3] Zhao Q., Fan Y., Kang B. (2017). A joint precoding scheme for indoor downlink multi-user MIMO VLC systems. Opt. Commun..

[4] Pham T.V., Pham A.T. (2019). Coordination/Cooperation Strategies and Optimal Zero-Forcing Precoding Design for Multi-User Multi-Cell VLC Networks. IEEE Trans. Commun..

[5] Duong S.T., Pham T.V., Nguyen C.T., Pham A.T. (2021). Energy-Efficient Precoding Designs for Multi-User Visible Light Communication Systems With Confidential Messages. IEEE Trans. Green Commun. Netw..

[6] Yu Z., Baxley R.J., Zhou G.T. Multi-user MISO broadcasting for indoor visible light communication. Proceedings of the ICASSP 2013—2013 IEEE International Conference on Acoustics, Speech and Signal Processing.

[7] Shen H., Xu W., Zhao K., Bai F., Zhao C. (2019). Non-Alternating Globally Optimal MMSE Precoding for Multiuser VLC Downlinks. IEEE Commun. Lett..

[8] Sun Z.G., Yu H.Y., Tian Z.J., Zhu Y.J. (2018). Linear Precoding for MU-MISO VLC Systems With Noisy Channel State Information. IEEE Commun. Lett..

[9] Sifaou H., Kammoun A., Park K.H., Alouini M.S. (2017). Robust Transceivers Design for Multi-Stream Multi-User MIMO Visible Light Communication. IEEE Access.

[10] Ma H., Lampe L., Hranilovic S. (2015). Coordinated Broadcasting for Multiuser Indoor Visible Light Communication Systems. IEEE Trans. Commun..

[11] Boyd S., Vandenberghe L. (2004). Convex Optimization.

[12] Dimitrov S., Haas H. (2015). Principles of LED Light Communications.

[13] Pascual-Iserte A., Pérez-Neira A.I., Palomar D.P., Lagunas M.A. (2006). A Robust Maximin Approach for MIMO Communications with Imperfect Channel State Information based on Convex Optimization. IEEE Trans. Signal Process..

[14] Sturm J.F. Using SEDUMI, a Matlab Toolbox for Optimization Over Symmetric Cones. https://sedumi.ie.lehigh.edu/.

[15] Bengtsson M., Ottersten B., Godara L.C. (2002). Optimum and Suboptimum Transmit Beamforming. Handbook of Antennas in Wireless Communications.

[16] Grant M., Boyd S. CVX: Matlab Software for Disciplined Convex Programming, Version 2.2; 2020. http://cvxr.com/cvx.

[17] Grant M., Boyd S., Blondel V., Boyd S., Kimura H. (2008). Graph implementations for nonsmooth convex programs. Recent Advances in Learning and Control.

